# Bioinformatic Analysis of *Leishmania donovani* Long-Chain Fatty Acid-CoA Ligase as a Novel Drug Target

**DOI:** 10.4061/2011/278051

**Published:** 2011-07-19

**Authors:** Jaspreet Kaur, Rameshwar Tiwari, Arun Kumar, Neeloo Singh

**Affiliations:** Drug Target Discovery & Development Division, Central Drug Research Institute (CSIR), Chattar Manzil Palace, Lucknow 226001, India

## Abstract

Fatty acyl-CoA synthetase (fatty acid: CoA ligase, AMP-forming; (EC 6.2.1.3)) catalyzes the formation of fatty acyl-CoA by a two-step process that proceeds through the hydrolysis of pyrophosphate. Fatty acyl-CoA represents bioactive compounds that are involved in protein transport, enzyme activation, protein acylation, cell signaling, and transcriptional control in addition to serving as substrates for beta oxidation and phospholipid biosynthesis. Fatty acyl-CoA synthetase occupies a pivotal role in cellular homeostasis, particularly in lipid metabolism. Our interest in fatty acyl-CoA synthetase stems from the identification of this enzyme, long-chain fatty acyl-CoA ligase (LCFA) by microarray analysis. We found this enzyme to be differentially expressed by *Leishmania donovani* amastigotes resistant to antimonial treatment. In the present study, we confirm the presence of long-chain fatty acyl-CoA ligase gene in the genome of clinical isolates of *Leishmania donovani* collected from the disease endemic area in India. We predict a molecular model for this enzyme for *in silico* docking studies using chemical library available in our institute. On the basis of the data presented in this work, we propose that long-chain fatty acyl-CoA ligase enzyme serves as an important protein and a potential target candidate for development of selective inhibitors against leishmaniasis.

## 1. Introduction

Leishmaniasis is a disease caused by protozoan parasites of the *Leishmania *genus. Visceral leishmaniasis (VL), also known as kala-azar, is the most severe form of leishmaniasis (http://www.dndi.org/diseases/vl.html). With no vaccine in sight, treatment for kala-azar relies primarily on chemotherapy [1]. 

Phylogenetics suggests that *Leishmania* is relatively early branching eukaryotic cells and their cell organization differs considerably from that of mammalian cells [2, 3]. Hence, the biochemical differences between the host and parasite can be exploited for identification of new targets for rational drug design. It is also imperative that the probability of developing drug resistance should be less with these targets. This can be achieved by targeting an essential cellular process, which has the pressure to remain conserved and cannot be bypassed by using alternative pathway.

One interesting target which emerged from our microarray experiments [4] was long-chain fatty acid-CoA ligase (EC 6.2.1.3) (GenBank Accession No. XM_001681734), a key enzyme involved in the metabolism of fatty acids in all organisms [5–9]. Fatty acyl-CoA has multiple roles involved in protein transport [10, 11], enzyme activation [12], protein acylation [13], cell signaling [14], transcriptional regulation [15], and particularly *β*-oxidation and phospholipid biosynthesis. Especially in *Leishmania*, long-chain fatty acids are predominant precursors of total lipid composition (the combination of phospholipids, sphingolipids, and ergosterol). Long-chain fatty acyl-CoA ligase is critical enzyme processing long-chain fatty acid acylation which is essential for lysophosphatidylinositol (lyso-PI) incorporation into glycosyl phosphatidylinositols (GPIs) [16, 17]. These GPIs-anchors are the major surface virulent factors in *Leishmania* and have received considerable attention [18]. *De novo* sphingolipid biosynthesis starts with the condensation of serine and the product of long-chain fatty acyl-CoA ligase. *L. major* preferentially incorporates myristoyl-CoA (C14) over palmitoyl-CoA (C16) into their long-chain base [19, 20]. This selection of specific long-chain fatty acyl-CoA reflects the presence of myristoyl-specific long-chain fatty acyl-CoA ligase in *Leishmania* [21]. 

Gaining new knowledge on fatty acid metabolism will not only provide fundamental insight into the molecular bases of *Leishmania* pathogenesis but also reveal new targets for selective drugs. Enzymes involved in fatty acid and sterol metabolism have been shown to be important pharmaceutical targets in *Leishmania* and other kinetoplastida [22]. Triacsin C, a specific inhibitor of long-chain fatty acyl-CoA synthetase, was shown to have an inhibitory effect on the growth of *Cryptosporidium parvum in vitro* [23]. 

Four fatty acyl-CoA synthetases have been described previously in *Trypanosoma brucei*, displaying different chain-length specificities [24, 25]. The whole genome sequence of three *Leishmania* spp. (*L. major*, *L. infantum,* and *L. braziliensis*) has been sequenced, and the availability of putative long-chain fatty acyl-CoA ligase genes was present in all three *Leishmania* spp. at chromosome 13, which would be required for initiation of *β*-oxidation and fatty acid metabolism. 

In the present study we confirm the presence of long-chain fatty acyl-CoA ligase gene in *Leishmania donovani *clinical isolate collected from, the state of Bihar India [26–29], which alone accounts for 50% of the total burden of visceral leishmaniasis worldwide [30]. Further progress in the understanding of this enzyme is likely to be achieved through the whole genome sequence (WGS) project of these clinically important isolates [26–29], underway in our laboratory (http://www.leishmaniaresearchsociety.org/).

## 2. Material and Methods

### 2.1. Collection of Clinical Isolates

The clinical isolates of *L. donovani* were collected from two kala-azar patients selected from Muzaffarpur, Bihar. The criterion for visceral leishmaniasis diagnosis was the presence of Leishman-Donovan (LD) bodies in splenic aspirations performed, which was graded to standard criteria [30]. Response to sodium antimony gluconate (SAG) treatment was evaluated by repeating splenic aspiration at day 30 of treatment. Patients were designated as antimonial responsive (*L. donovani* isolate 2001) based on the absence of fever, clinical improvement with reduction in spleen size, and absence of parasites in splenic aspirate while patients who showed presence of parasites in splenic aspiration were considered to be antimonial unresponsive (*L. donovani* isolate 39) [26–29].

### 2.2. Sample Collection and Nuclear DNA Isolation

*L. donovani* isolates 2001 (SAG-sensitive) and 39 (SAG-resistant) used in the present study, were maintained in culture as described previously in [26–29]. For nuclear DNA isolation 10–15 mL log-phase culture was taken and centrifuged at 5,000 rpm for 8 min at 4°C. The supernatant was decanted; cell pellet was resuspended in 3–6 mL NET buffer and centrifuged at 5,000 rpm for 8 min at 4°C. The supernatant was discarded, and the pellet was redissolved in 750 *μ*L NET buffer, 7.5 *μ*L proteinase K (10 mg/mL stock) (MBI, Fermentas, Cat No. EO0491), and 50 *μ*L of 15% sarkosyl. Sample was incubated at 37°C overnight for proteinase K activity. The cell lysate was centrifuged at 18,000 rpm for 1 hr at 4°C. The supernatant containing nuclear DNA was transferred to a fresh tube and given RNase treatment (20 *μ*g/mL) (MBI, Fermentas, Cat No. EN0531) at 37°C for 30 min. DNA was extracted first with one volume phenol/chloroform/isoamyl alcohol (25 : 24 : 1) and finally with chloroform. Nuclear DNA was precipitated with 2.5 volumes of prechilled absolute ethanol, dissolved in nuclease-free water and stored at 4°C for future use.

### 2.3. Primer Design, PCR Amplification, and Sequencing of Long-Chain Fatty Acyl-CoA Ligase Gene

PCR amplification was carried out using *Pfu* DNA polymerase (MBI, Fermentas, Cat No. EP0501). Reactions were carried out in a Perkin Elmer GeneAmp PCR system with 2001 nuclear DNA (10–50 ng) as template. The following oligonucleotide primers were designed on the basis of available gene sequence of *L. major* (GenBank Accession No. XM_001681734): forward primer: 5′GGGCCATATGCTGCAGCG 3′ (18 mer) and reverse primer: 5′GGCCTCGAGCTAAAACAAATCATCG3′ (25 mer). The amplification conditions were initial denaturation at 95°C for 10 min, denaturation at 95°C for 30 sec, annealing at 65°C for 1 min, extension at 72°C for 2 min, and final extension at 72°C for 10 min; 30 cycles. The PCR product was purified from agarose gel using MBI Fermentas DNA Extraction kit (MBI, Fermentas, Cat No. K0513) and further for DNA sequencing by Bangalore Genei, India.

### 2.4. Characterization of Long-Chain Fatty Acyl-CoA Ligase Gene

*L. donovani *nuclear DNA (16 *μ*g for each reaction) of two different clinical isolates, drug (SAG) sensitive 2001 and drug (SAG) resistant 39, were digested with 40-unit three different restriction enzymes (*Pvu*II, *Bam*HI, and *Xho*I), which were cut overnight and separated on 0.8% agarose gel by electrophoresis at 50 V. In order to improve transfer efficacy, DNA in agarose gel was treated with 0.25 N HCl for 15 min (partial depurination), rinsed with autoclaved water 3x, and treated with 0.4 N NaOH (breaking backbone at depurinated region) for 30 min. DNA was transferred to nylon membrane by conventional downward capillary transfer method for 5 h using 3 mm Whatman paper wick [8]. The efficiency of transfer was assessed by visualizing DNA by methylene blue staining. After transfer on nylon membrane the DNA was neutralized in 0.5 M Tris (pH 7.4), 1.5 M NaCl, 2x for 5 min at room temperature. The membrane was then washed in 2X SSC, 2x for 15 min. Nylon membrane was incubated with 2.5 mL of prehybridization buffer (0.6 M NaCl, 0.5 M Tris-HCl (pH 7.5), 0.008 M EDTA, 1% sodium pyrophosphate, 0.2% SDS, and 50 *μ*g/mL heparin) and incubated in a hybridization oven at 65°C for 2 h. Radioactive probe was prepared by labeling 25 ng of the DNAs with [*α*-^32^P] dCTP by random priming method (BRIT/BARC, India) and purified using a desalting column (sephadex G-50). The radioactivity was checked with a Geiger Muller Counter (dosimeter) and stored at −20°C. The probe was added to the prehybridization buffer and incubated at 65°C overnight in hybridization oven. Membrane was washed twice with 2X SSC, 0.1% SDS (15 min each) at 65°C and then washed with 2X SSC, 0.1% SDS for 30 min at 65°C to reduce background signals. Hybridized membrane was layered over a wet Whatman paper sheet to soak extra solution and covered with Saran Wrap (cellophane paper) and exposed to X-ray film. After 4–18 h exposure in an exposure cassette at −70°C, X-ray film was developed for analysis.

### 2.5. Phylogenetic Analysis

The amino acid sequence of *Leishmania* long-chain fatty acyl-CoA ligase, obtained from our microarray experiments [4], was compared with sequences available in GeneDB ORTHOMCL4080 database (http://www.genedb.org/) to identify the nearest ortholog of this sequence in kinetoplastida. Multiple sequence alignments were performed using Clustal W version 1.8 (http://www.ebi.ac.uk/clustalw) and T-cofee [31]. To calculate evolutionary distances of kinetoplastida long-chain fatty acyl-CoA ligases with human acyl CoA synthetases (ACSs) [32], phylogenetic dendrograms were constructed by neighbor-joining method and tree topologies were evaluated by performing bootstrap analysis of 1000 data sets using MEGA 3.1 (Molecular Evolutionary Genetics Analysis) [33]. All 26 human ACSs amino acid sequences were selected [32], along with their transcript variants which are aligned with different long-chain fatty acyl-CoA ligase ortholog present in kinetoplastida family, to define the clade difference with *Trypanosome* and *Leishmania* long-chain fatty acyl-CoA ligase, and human acyl-CoA synthetases.

### 2.6. Homology Modeling of *Leishmania* Long-Chain Fatty Acyl-CoA Ligase

The amino acid sequence of *Leishmania* long-chain fatty acyl-CoA ligase was retrieved from the NCBI database (GenBank Accession No. XM_001681734). It was ascertained that the 3D structure of *Leishmania* long-chain fatty acyl-CoA ligase protein was not available in Protein Data Bank (PDB); hence, the present exercise of developing the 3D model of this protein was undertaken. cBLAST (http://www.ncbi.nlm.nih.gov/Structure/cblast/cblast.cgi) and PSI-BLAST search was performed against PDB with the default parameter to find suitable templates for homology modeling. The sequence alignment of *Leishmania *long-chain fatty acyl-CoA ligase and respective templates was carried out using the CLUSTALW (http://www.ebi.ac.uk/clustalw) and MODELLER9V8 programs [34, 35]. The sequences that showed the maximum identity with high score and lower e-value were used as a reference structure to build a 3D model. 

The retrieved sequences of *Thermus thermophilus *(PDB Accession Code: 1ULT, 1V25, 1V26) [36] and *Archaeoglobus fulgidus *(PDB Accession Code: 3G7S) long-chain fatty acyl-CoA ligases served as template for homology modeling based on its maximum sequence similarity to *Leishmania *long-chain fatty acyl-CoA ligase. The alignment was manually refined at some loops region of the templates. The resulting alignment was used as an input for the automated comparative homology modeling for generating 3D model structure of *Leishmania *long-chain fatty acyl-CoA ligase. The academic version of MODELLER9V8 was used for model building. The backbones of core region of the protein were transferred directly from the corresponding coordinates of templates. Side chain conformation for backbone was generated automatically. Out of 50 models generated by MODELLER, the one with the best DOPE score, minimum MOF (Modeller Objective Function), and best VARIFY 3D profile was subjected to energy minimization. In order to assess the stereochemical qualities of 3D model, PROCHECK analysis [37] was performed and Ramachandran plot was drawn.

## 3. Results

### 3.1. Metabolism of Long-Chain Fatty Acyl-CoA Ligase Enzyme

Three types of fatty acyl CoA ligase have been defined with respect to the length of the aliphatic chain of the substrate: short (SC-EC 6.2.1.1), medium (MC-EC 6.2.1.2), and long-chain (LC-EC 6.2.1.3) fatty acyl-CoA ligase. These utilize C2-C4, C4-C12, and C12-C22 fatty acids as substrates, respectively [9]. Fatty acid activation step involves the linking of the carboxyl group of the fatty acid through an acyl bond to the phosphoryl group of AMP. Subsequently, a transfer of the fatty acyl group to the sulfhydryl group of CoA occurs, releasing AMP [38–40]. This magnesium-dependent two-step acylation of fatty acid by fatty acyl CoA synthetases was defined as unidirectional Bi Uni Uni Bi Ping-Pong mechanism [36, 39]. 

Genome analysis suggests that *L. major* oxidizes fatty acids via *β*-oxidation in two separate cellular compartments: the glycosome and mitochondria [41]. An argument for the involvement of glycosome in lipid metabolism is the fact that in each of three trypanosomatid genomes three genes called half ABC transporters (GATI 1-3) have been found identical with peroxisomal transporters involved in fatty acid transport. In *T. brucei*, it was conformed that these transporters are associated with glycosomal membrane [42]. These transporters might be coupled with fatty acyl-CoA ligase in glycosome, which can provide activated form of fatty acids to these transporters like oleoyl-CoA, and also other acylated fatty acids.

In *T. brucei,* little *β*-oxidation was observed in mitochondria. However, *T. brucei* contains at least two enzymes involved in *β*-oxidation of fatty acid (2-enoyl-CoA hydratase and hydroxyacyl-dehydrogenase, encompassed in a single protein) with glycosome localization [43]. The presence of a PTS (Peroxisomal Targeting Sequence) on *T. brucei* and *T. cruzi* carnitine acetyl transferase, catalysing the last peroxisomal step in fatty acid oxidation, suggests that the major *β*-oxidation processes are situated in glycosomes [44]. In *L. donovani*, one of the *β*-oxidation enzyme 3-hydroxyacyl-CoA dehydrogenase has been localized to glycosomes [45]. The hypothetical localization of *Leishmania* long-chain fatty acyl-CoA ligase was predicted in mitochondria or glycosome but, with the reference of other organisms, the specialized localization of specific long-chain fatty acyl-CoA ligase family protein needs to be taken into account in future. 

As mentioned in a previous study *β*-oxidation has been found to be unregulated in *Leishmania*'s amastigotes then in promastigote stage [46–48]. This specialized increase was described so that, in infectious stage, energy requirement was supplemented to utilize fatty acid as carbon and energy source rather than glucose [47]. Long-chain fatty acyl-CoA ligase is the key enzyme involved in *β*-oxidation of fatty acids, and its compartmentation in glycosome supports a strong evidence of the involvement of this enzyme in cellular biogenesis and its importance at particular stage of *Leishmania* life cycle. In the same way upregulation of long-chain fatty acyl-CoA ligase with combination of other enzymes involved in fatty acid catabolism might play a crucial role in cell survival at infectious stage of *Leishmania,* and these analyses must be supplemented with experimental biology.

### 3.2. Characterization of *Leishmania* Long-Chain Fatty Acyl-CoA Ligase Gene

**Figure 1:**
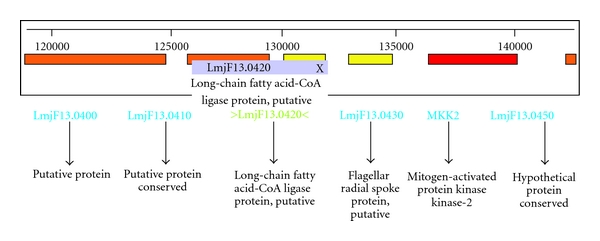
Graphical representation of long-chain fatty acyl-CoA ligase (LCFA) gene (in Artemis) on chromosome 13 of *Leishmania major*.

**Figure 2:**
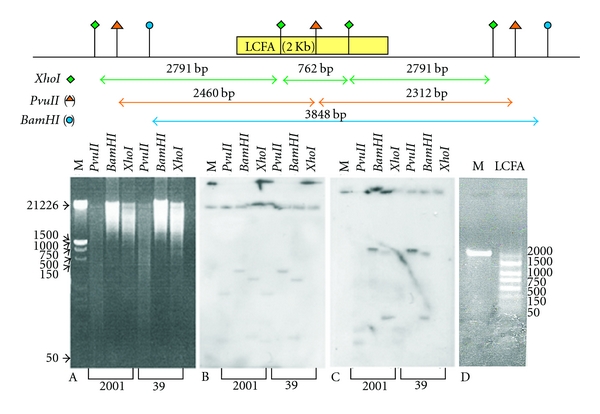
Determination of long-chain fatty acyl-CoA ligase gene copy number. The nuclear DNA of *L. donovani *2001 and 39 promastigotes was isolated and 16 *μ*g was digested with different restriction enzymes. (A) Nuclear DNA digest stained with ethidium bromide (B) Southern blot of “A” with *α*-tubulin gene probe. (C) Southern blot of “A” with long-chain fatty acyl-CoA ligase probe. (D) PCR amplification of long-chain fatty acyl-CoA ligase gene (M: Marker, LCFA: 2010 bp of long-chain fatty acyl-CoA ligase gene).

The presence of *L*.* donovani *long-chain fatty acyl-CoA ligase gene in the clinical isolates was ascertained by PCR amplification. The putative long-chain fatty acid-CoA ligase gene of *L. major* is present in the *Leishmania *Genome Databank (http://www.genedb.org/) on chromosome 13 (Figure 1). Specific 2010 bp size amplified product was obtained, showing the presence of long-chain fatty acyl-CoA ligase gene in the *L. donovani* clinical isolate (Figure 2(D)). The amplified product was sequenced and confirmed to be long-chain fatty acid-CoA ligase gene by performing NCBI-BLAST identity with *L. major* gene. NCBI-BLAST result showed 96% sequence similarity and 1% gaps with *L. major* long-chain fatty acyl-CoA ligase gene (GenBank Accession No. XM_001681734). The starting 18 nucleotides and 19 nucleotides from the end sequence were missed due to direct amplified product sequencing. These nucleotides were collected from its maximum similar *L. major* long-chain fatty acyl-CoA ligase sequence (GenBank Accession No. XM_001681734).

For the determination of long-chain fatty acid-CoA ligase gene copy number, nuclear DNA from the *L. donovani* clinical isolates (2001, 39) was digested with various restriction enzymes. The restriction map was designed from the complete putative long-chain fatty acyl-CoA ligase gene and the flanking region present in chromosome 13 of *L. major* (Figure 2). Southern hybridization was performed using the 2010 bp long-chain fatty acid-CoA ligase gene PCR product as probe (Figure 2(C)). The same blot was also probed with alpha tubulin gene probe as an internal control, showing equal loading (Figure 2(B)). Complete digestion resulted in a single copy within the *L. donovani *genome, as *Bam*HI enzyme showed only one band of approximately 3848 bp, except *Pvu*II which was cut once into the gene sequence and *Xho*I which was cut twice into the gene sequence, which exhibited two and three hybridizing bands, respectively. The results showed that long-chain fatty acid-CoA ligase is present as a single copy gene in the *L. donovani* genome. The restriction pattern also verifying the restriction pattern of *L. donovani *and *L. major *long-chain fatty acyl CoA ligase coding region is almost the same.

### 3.3. Identification of Conserved Domains and Structure-Function Correlation in *Leishmania* Long-Chain Fatty Acyl-CoA Ligase

**Figure 3:**
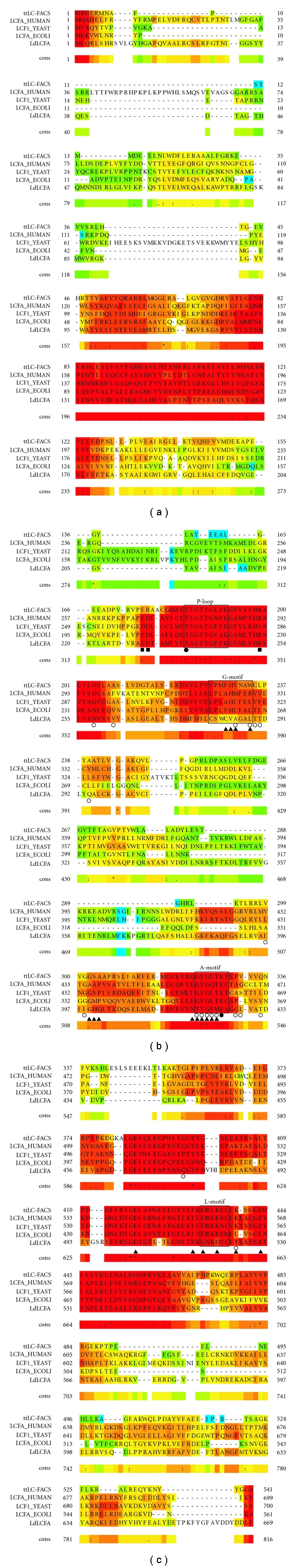
Amino acid sequence alignments of long-chain fatty acyl-CoA synthetases. The amino acid sequence of *Leishmania* LCFA (LdLCFA) was aligned with LC-FACS from *T. thermophilus* (ttLC-FACS, Q6L8FO), human (LCFA_HUMAN, P41215), yeast (LCF1_YEAST, P30624), and *E. coli* (LCFA_ECOLI, P29212). The *boxed areas *denoted with *bold letters *correspond to conserved motifs of long-chain fatty acyl-CoA ligase: G, A, and L motifs as well as the P-loop. *Filled squares*, *open circles*, *filled circles*, and *filled triangles *indicate residues believed to be involved in dimer formation, fatty acid binding, magnesium ion binding, and adenylate binding, respectively.

*Leishmania* long-chain fatty acyl-CoA ligase gene was translated from full length ORF on the basis of its nucleic acid sequence. Long-chain fatty acyl-CoA synthetase from *T. thermophilus*, yeast, and *E. coli* and all 26 distinct human acyl-CoA synthetases were subjected to phylogenetic analysis to facilitate the evaluation of conserved motif with relationship of reference *Leishmania* long-chain fatty acyl-CoA ligase amino acid sequence. The amino acid sequence of *Leishmania* long-chain fatty acyl-CoA ligase (*Ld*LCFA) was aligned with LC-FACS from *T. thermophilus* (*Tt*LC-FACS,1ultA), human (LCFA_HUMAN, P41215), yeast (LCF1_YEAST, P30624), and *E. coli* (LCFA_ECOLI, P29212) on the basis of PSI-BLAST (Figure 3). However the overall similarity of *Leishmania* long-chain fatty acyl-CoA ligase (*Ld*LCFA) with other fatty acyl-CoA synthetases family proteins is low, about 17% with *Tt*LC-FACS, 15% with LCFA_HUMAN, 14% with LCF1_YEAST, and 13% with LCFA_ECOLI. Based on the crystal structure of *Tt*LC-FACS and alignment with other long-chain fatty acyl-CoA synthetases [36], the amino acid sequence of *Leishmania* long-chain fatty acyl-CoA ligase shows conserve region corresponding to the linker (L), adenine (A), and gate (G) motifs as well as the P-loop, the phosphate-binding site. Previous studies [32, 36] put forward different motifs which can give insight to enhance our understanding of predicted structure-function relationships in *Leishmania*. P-loop is the Motif I which is also known as AMP-binding domain found in a close proximity to the adenosine moiety and helps to maintain the substrate in the proper orientation. The consensus sequence of Motif I, [Y,F]TSG[T,S]TGXPK shows high level of conservation with respect to *Leishmania* long-chain fatty acyl-CoA ligase, that is, 237-FTAGTTGPPK-246. Motif II contains the L-motif (432-DRLKDL-437) that acts as a linker between the large N-terminal domain and the smaller C-terminal domain in TtLC-FACS. The linker region is thought to be critical for catalysis function as it facilitates a conformational change upon ATP binding that permits subsequent binding of the fatty acyl and/or CoA substrates. In* Leishmania* long-chain fatty acyl-CoA ligase, this linker region (517-GNKDVL-522) is less similar compared with other organisms and is likely to be critical in enzyme activity. Motif III was found to be in all acyl CoA synthetases and a part of A-motif (adenine motif). This region has been described as an ATP/AMP-binding domain in other acyl-CoA synthetases [49–51]. The conserved consensus sequence of A-motif is YGXTE, a highly conserved motif with respect to *Leishmania* long-chain fatty acyl-CoA ligase region, that is, YGFME. From the crystal structure of *Tt*LC-FACS, it was proposed that Y324 was an adenine-binding residue [42] and also conserved throughout all organisms including *Leishmania*. The crystal structure of *S. enterica* acetyl-CoA synthetase revealed that the glutamate residue of A-motif is positioned near oxygen O1 of the AMP phosphate [52]. This region was predicted to be involved in substrate binding or stabilization, conserved in *Leishmania* long-chain fatty acyl-CoA ligase also. Motif IV comprises the first five residues of the nine-amino acid G-(or gate) motif (226-VPMFHVNAW-234) of ttLC-FACS (36), showing less sequence similarity with *Leishmania *long-chain fatty acyl-CoA ligase (281-CSWCVAGAL-289). From the crystal structure of *Tt*LC-FACS, it was proposed that the indole ring of W234 acts as a gate and blocks the entry of fatty acids into its substrate binding tunnel unless ATP is first bound, resulting in a conformational change that swings the gate open (36). However, a tryptophan residue corresponding to W234 was not found in any *Leishmania*, human, yeast, and *E. coli* fatty acyl-CoA synthetase sequences. In contrast, although no highly conserved sequences were identified, a corresponding gate residue may be located elsewhere in the structure of *Leishmania* long-chain fatty acyl-CoA ligase.

**Figure 4:**
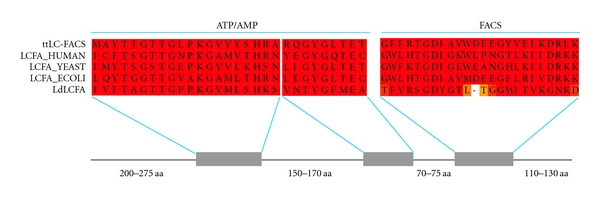
Domain organization of amino acid sequence alignments of ATP-AMP and fatty acyl CoA synthetase (FACS) motif from *T. thermophilus *(ttLC-FACS), human (LCFA_HUMAN), yeast (LCF1_YEAST), *E. coli *(LCFA_ECOLI), and *Leishmania* (L LCFA).

The fatty acyl-CoA synthetases are part of a large family of proteins referred to as the ATP-AMP-binding proteins. A common feature of enzymes in this family is that they all form an adenylated intermediate as part of their catalytic cycle. This group of enzymes is diverse in catalyzing the activation of a wide variety of carboxyl-containing substrates, including amino acids, fatty acids, and luciferin. Sequence comparison of members of the ATP-AMP-binding protein family has identified two highly conserved sequence elements, [53] Y[T]S[GTTG]X[PKGV]*⋯*G[YG]XT[E] (the bracket shows the conserved sequence in *Leishmania* long-chain fatty acyl-CoA ligase), which encompass the ATP-AMP signature motif (Figure 4). 

In fatty acyl-CoA synthetases family proteins, there was a third sequence element defined as FACS signature motif that was less conserved and partially overlaps the FACS signature motif, which is involved in both catalysis and specificity of the fatty acid substrate [54]. There are a number of notable features within the FACS signature motif: (i) this region contains two invariant glycine residues (at positions 2 and 7) and a highly conserved glycine at position 16, *Leishmania* long-chain fatty acyl-CoA ligase shares glycine residue with other FACSs at position 7 and 16 but Tyr instead of Gly was found in position 2. (ii) This region contains additional six residues that are invariant in the fatty acyl-CoA synthetases: W[3], T[6], D[8], D[22], R[23], and K[25], but in *Leishmania* long-chain fatty acyl-CoA ligase these residues are F[3], S[6], D[8], G[22], N[23], and D[25]. (iii) The residue in the fourth position is hydrophobic and is a leucine, a methionine, or phenylalanine. However, in *Leishmania *long-chain fatty acyl-CoA ligase hydrophobic residue valine was situated in position 4. (iv) This region of enzyme contains hydrophobic residues (leucine, isoleucine, or valine) at positions 4, 9, 18, 20, and 21. These residues, in addition to tryptophan or phenylalanine residues at position 3, may comprise part of a fatty-acid-binding pocket. All of these five conserved regions from FACS signature motif are having similarity among them except *Leishmania* long-chain fatty acyl-CoA ligase, with some variable regions. These less conserved regions in *Leishmania* long-chain fatty acyl-CoA ligase-FACS signature motif were predicted to adopt inconsistent specificity and catalytic activities of the fatty acid substrate compared to other fatty acyl CoA synthetases.

### 3.4. Phylogenetic Analysis of *Leishmania* Long-Chain Fatty Acyl-CoA Ligase and Human Acyl-CoA Synthetases Sequences

**Figure 5:**
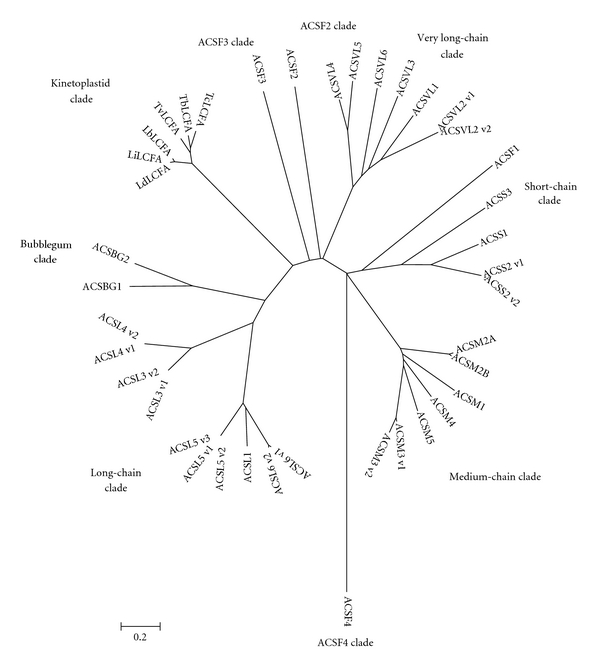
Phylogenetic trees based on human acyl CoA synthetases (ACSs) gene sequences [38] showing the relationship of all *Leishmania* long-chain fatty acyl-CoA ligase orthologs (Table 1), with their nearest phylogenetic relatives. Phylogenetic trees were constructed by the neighbour-joining method as well as the maximum likelihood method as implemented in MEGA4 software. Numbers at nodes are bootstrap values (ML/NJ; xx represents no bootstrap value in NJ tree where nodes differ in both dendrograms;—represents value <50). The bar represents 0.02 substitutions per alignment position. The bar represents substitutions per alignment position.

We performed phylogenetic analysis to infer evolutionary relationships of all available sequences from kinetoplastida long-chain fatty acyl-CoA ligases (Table 1) and human (host) ACSs family sequences. This experiment was performed to validate that the parasite enzyme is unquestionably different from the human enzyme, and this aspect merits further study to validate this enzyme as a drug target. We obtained comparable results using the neighbor-joining distance-based algorithm as well as maximum parsimony. We found 9 clades, including kinetoplastida clade (one set of six kinetoplastida long-chain fatty acyl-CoA ligase protein family) forming a clade with high bootstrap support (Figure 5). kinetoplastida clade was highly dissimilar and distinct from all ancestral nodes with other human ACSs family proteins and showing distinctiveness of kinetoplastida long-chain fatty acyl-CoA ligases, including *Leishmania* long-chain fatty acyl-CoA ligase. This divergence of *Leishmania* long-chain fatty acyl-CoA ligase with respect to the homologous human enzymes may be an important protein as a potential target candidate for chemotherapeutic antileishmanial drugs. 

### 3.5. Homology Modeling of *Leishmania* Long-Chain Fatty Acyl-CoA Ligase Protein

**Figure 6:**
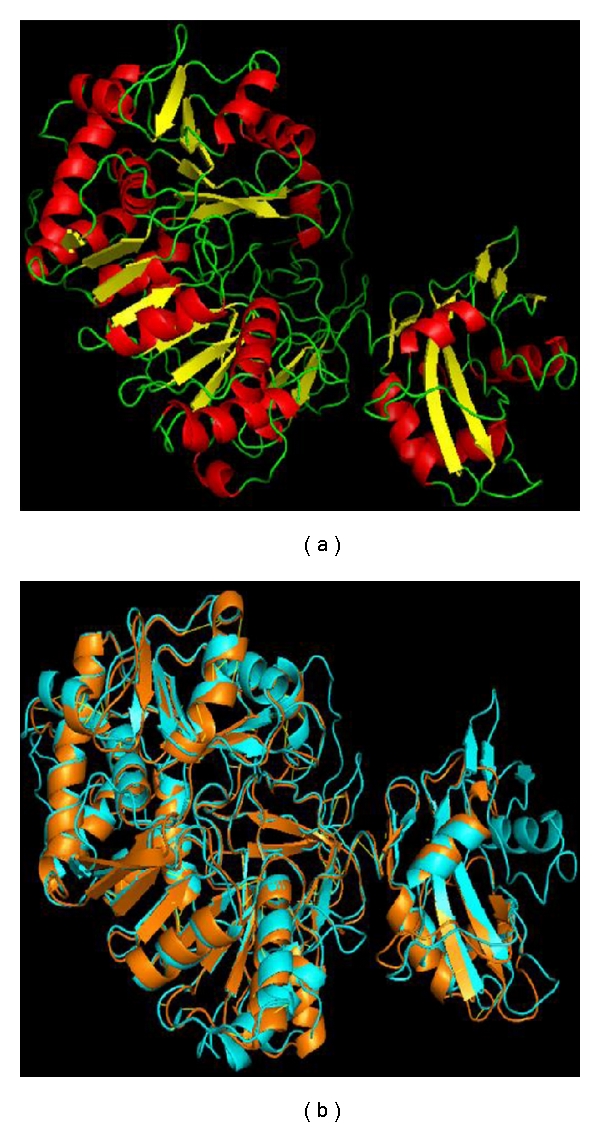
*Leishmania *long-chin fatty acyl-CoA ligase model. (a) The larger left hand-side domain is the N-terminal domain and the smaller one is the C-terminal domain which is connected by a linker chain. (b) Superposition of the modeled structure of *Leishmania* long-chain fatty acyl-CoA ligase (Orange) with the crystal structure of the *T. thermophilus* long-chain fatty acyl-CoA synthetase (PDB code: 1ult A) (Blue).

**Figure 7:**
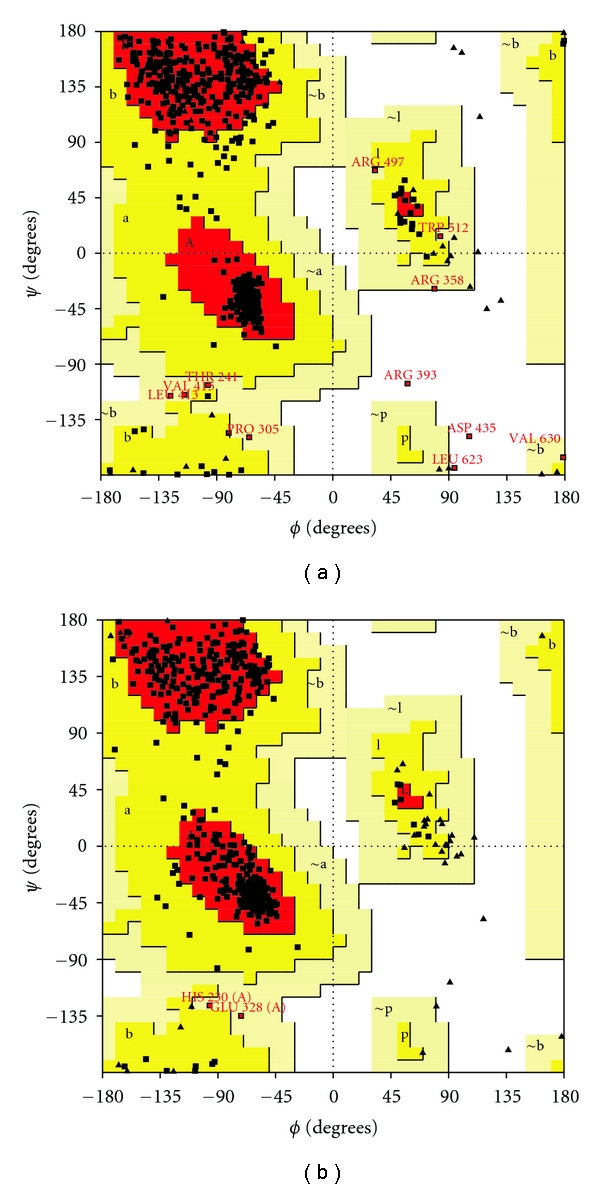
Ramachandran plot of (a) modeled structure *Leishmania* long-chain fatty acyl-CoA ligase (b) and the crystal structure of the Tt0168 (PDB code: 1ult A).

The backbone root-mean-square-deviation (RMSD) values between final model and template crystal structure used are 1.04 Å with *Thermus thermophilus *(PDB Accession Code: 1ULT, 1V25, 1V26) and 1.40 Å with *Archaeoglobus fulgidus *(PDB Accession Code: 3G7S) long-chain fatty acyl-CoA ligase. Small RMSD can be interpreted as structures share common structural homology and the generated structure is reasonable for structural similarity analysis (Figure  6). The final modeled structure of *Leishmania* long-chain fatty acyl-CoA ligase was evaluated for overall quality using available analyses procedures. These analysis compare specific properties of the model with those of known high-quality protein structures using programs like PROCHECK, Verify3D, and WHATIF (Table  2). An important indicator of the stereochemical quality of the model is distribution of the main chain torsion angles phi and psi in Ramachandran plot (Figure  7). The plot clearly shows the vast majority of the amino acids in a phi-psi distribution consistent with right *α*-helices, and the remaining fall into beta configuration. Only three residues fall outside the allowed regions. Plots comparison shows that the structure is reasonable overall because the space distribution for the homology-modeled structure was similar to the X-ray structure of the *Thermus thermophilus *long-chain fatty acyl-CoA ligase (PDB Accession Code: 1ULTA). The results showed that our modeled structure was reasonably good at that much less sequence identity.

## 4. Discussion

Earlier during the course of work, microarray analysis was performed on the same clinical *L. donovani* isolates (2001 and 39) in order to identify differential gene expression [4]. Out of all genes found differentially expressed, significant upregulation of long-chain fatty acyl-CoA ligase gene in SAG unresponsive clinical isolate [33] was found to be intracellular amastigote specific and has confirmed the involvement of long-chain fatty acyl-CoA ligase in resistance. Similarly, it has been proven before that the rate limiting enzyme, long-chain fatty acyl-CoA ligase of *β*-oxidation, was found to be upregulated in amastigotes derived from cloned line of *L. donovani* ISR because, during late stages of differentiation, the parasites shift from glucose to fatty acid oxidation as the main source of energy, and thereby there is increase in enzyme activity associated with *β*-oxidation capacity [47, 48]. Early *in vivo* studies showed that enzymatic activities associated with *β*-oxidation of fatty acids were significantly higher in *L. mexicana *amastigotes [47]. Additionally microarray experiments with intracellular amastigotes hybridized onto Affymetrix Mouse430_2 GeneChips showed that several genes involved in fatty acid biosynthesis pathway were found to be upregulated [55]. Presently studies are ongoing in our laboratory on microarray analysis using intracellular amastigotes hybridized to Affymetrix GeneChip human genome U133 Plus 2.0 array which will further yield useful information towards the fatty acid/lipid metabolism within this clinical isolate. A very recent study by Yao et al., 2010, on differential expression of plasma membrane proteins in logarithmic versus metacyclic promastigotes of *L. chagasi *has also identified long-chain fatty acyl-CoA synthetase [56].

As mentioned before, long-chain fatty acid-CoA ligase is present in both prokaryotes and eukaryotes. This divergence of *Leishmania* long-chain fatty acyl-CoA ligase with respect to the homologous human enzymes may be an important protein as a potential target candidate for chemotherapeutic antileishmanial drugs. Many differences exist between host and parasite pertaining to the structure and arrangement of this enzyme. However, *Leishmania* has significant divergence and adaptation to specific environmental conditions between its two life stages, in the insect vector and human host. This can affect the parasites metabolic machinery in terms of presence of certain pathways, their subcellular localization and expression at different developmental stages, and the interplay between scavenging and synthesis of key metabolites. It has been argued previously [57] that successful targets for metabolic intervention are most likely to be found among enzymes exerting strong control of flux through metabolic pathways. These control points are likely to be species and development dependent. Even if a unique or highly divergent enzymatic process is found in the parasites, this does not necessarily mean it can be developed as a target for useful inhibitors. On the other hand, enzymes that are present in both the parasites and their animal hosts will often differ sufficiently in their sequence for inhibitors to be specific. Finally, even orthologous enzymes functioning in the same pathway and in the same subcellular compartment of the parasites may have different inhibitor binding properties, leading to variability in the effectiveness and specificity of inhibitors targeting any particular enzyme.

The detection of the long-chain fatty acid-CoA ligase gene in the genome of *L. donovani* clinical isolate, in the present study, deserves a full exploration with respect to its potential as a drug target. Changes in membrane lipids/deficiency of certain fatty acids and disease association have been documented [34, 58]. Modulation of enzymes involved in lipid synthesis and of others possibly involved in cell wall metabolism may modify access of drug to the plasma membrane. Moreover, our microarray experiment indicated that this enzyme was amastigote specific making it all the more important to study it further and test if it can be exploited as a validated drug target. We have also shown earlier in our laboratory [34] that modification of lipid composition on the plasma membrane of the parasite might have important implications towards generating susceptibility/resistance to antileishmanial drugs. As this enzyme stipulates several important cellular processes in *Leishmania* like stage-specific expression [47, 48], host-parasite interaction [55], cell membrane composition [17, 18], phospholipid biosynthesis [16, 21], and drug resistance [4], the present study proposed further evaluation of *Leishmania* long-chain fatty acyl-CoA ligase as a candidate drug target.

## Figures and Tables

**Table 1:** Selected ortholog for *Leishmania donovani* long-chain fatty acyl-CoA ligase gene in kinetoplastida: ORTHOMCL4080 (http://www.genedb.org/).

Systematic ids	Organism	Product
LbrM13_V2.0240	*L. braziliensis *MHOM/BR/75/M2904	Fatty acid thiokinase (long chain), putative; acyl-CoA synthetase, putative; long-chain-fatty acid-CoA ligase protein, putative

LinJ13_V3.0300	*L. infantum *JPCM5	Fatty acid thiokinase (long chain), putative; long-chain-fatty acid-CoA ligase protein, putative; acyl-CoA synthetase, putative

LmjF13.0420	*L. major strain *Friedlin	Long-chain fatty acid-CoA ligase protein, putative; acyl-CoA synthetase, putative; fatty acid thiokinase (long chain), putative

Tb11.02.2070	*T. brucei *927	Long-chain fatty acid-CoA ligase protein, putative; fatty acid thiokinase (long chain), putative; acyl-CoA synthetase, putative

Tc00.1047053504089.40	*T. cruzi*	Long-chain fatty acid-CoA ligase protein, putative; acyl-CoA synthetase, putative; fatty acid thiokinase, long chain, putative

TvY486_1104610	*T. vivax*	Long-chain fatty acid-CoA ligase protein, putative

**Table 2:** Results of protein structure by PROCHECK and VERIFY 3D.

	*Leishmania* long-chain fatty acyl-CoA ligase	*T. thermophilus* long-chain fatty acyl-CoA synthetase (1ULTA)
Residues in most favoured regions	521 (87.9%)	405 (90.0%)
Residues in additional allowed regions	62 (10.5%)	43 (9.6%)
Residues in generously allowed regions	7 (1.2%)	2 (0.4%)
Residues in disallowed regions	3 (0.5%)	0 (0.0%)
Number of nonglycine and nonproline residues	593	450
Number of end-residues (excl. Gly and Pro)	2	155
Number of glycine residues (shown as triangles)	48	48
Number of proline residues	26	34
Total number of residues	669	687
Residues with Verify 3D Score >0.2	52.24%	96.63%
Errat overall quality factor	44.154	89.655
